# Large-scale real-life analysis of survival and usage of therapies in multiple myeloma

**DOI:** 10.1186/s13045-023-01474-w

**Published:** 2023-07-19

**Authors:** N. Lopez-Muñoz, G. Hernández-Ibarburu, R. Alonso, J. M. Sanchez-Pina, R. Ayala, M. Calbacho, C. Cuellar, M. T. Cedena, A. Jiménez-Ubieto, R. Iñiguez, M. Pedrera, J. Cruz, L. Meloni, D. Pérez-Rey, P. Serrano, J. de la Cruz, J. Martinez-Lopez

**Affiliations:** 1 Hematology Department, Hospital 12 de Octubre, CNIO, Complutense University, Madrid, Spain; 2 Biomedical Informatics Group, Universidad Politécnica de Madrid, Madrid, Spain; 3 Data Science Group, Hospital 12 de Octubre, Madrid, Spain; 4 TriNetX Europe NV, Sint-Martens-Latem, Belgium; 5 Research Institute imas12, Hospital 12 de Octubre, Madrid, Spain

**Keywords:** Multiple myeloma, Survival, Treatments, Real world data, TriNetX

## Abstract

Survival in multiple myeloma has improved significantly in recent years, especially in young patients. We reviewed the evolution of the survival of patients with MM in three groups based on age at MM diagnosis over three time periods between 1999 and 2020 at our 12 de Octubre Hospital institution (H12O). Then, to confirm our results, we used data from TriNetx, a global health research platform that includes patients from Europe to US. Finally, we analysed differences in the patterns of treatment between networks across the world. Kaplan‒Meier analysis was used to estimate survival probabilities, and between-group differences were tested using the log-rank test and hazard ratio. For patients from H12O, the median OS was 35.61, 55.59 and 68.67 months for the 1999–2009, 2010–2014 and 2015–2020 cohorts, respectively (*p* = 0.0001). Among all patients included in the EMEA network, the median OS was 20.32 months versus 34.75 months from 1999–2009 versus 2010–2014. The median OS from the 2010–2014 versus 2015–2020 time cohorts was 34.75 months versus 54.43 months, respectively. In relation to the US cohort, the median OS from before 2010 versus 2010–2014 was not reached in either time cohort and neither when comparing the 2010–2014 versus 2015–2019 time cohorts. Bortezomib is the most commonly used drug in the EMEA cohort, while lenalidomide is the most commonly used drug in the US cohort. This large-scale study based on real-world data confirms the previous finding that MM patients have increased their survival in the last two decades.

**Supplementary Information:**

The online version contains supplementary material available at 10.1186/s13045-023-01474-w.

**To the editor**,

Survival in multiple myeloma has improved significantly in recent years, especially in young patients. This is due to the introduction of new drugs with new mechanisms of action [1–5]. However, most of the available data have been obtained from clinical trials or local registries [6–8], both of which have included very limited populations. TriNetx is a global health research platform that provides researchers with access to health care organizations’ (HCOs’) electronic health records for research purposes.

In this study, we compared survival time since MM diagnosis in three groups based on age at MM diagnosis (younger than 65 years old, between the ages of 65 and 75 years old, and older than 75 years old) over three time periods: 1999–2009, 2010–2014 and 2015–2020. Finally, treatment patterns in the EMEA and US networks over the last 20 years (1999 to 2019) were analysed. This study retrospectively examined 703 patients diagnosed, between 1999 and 2020, with symptomatic MM (ICD-10-CM code C90.0) at a tertiary hospital in Spain, Hospital 12 de Octubre (H12O). Comparator cohorts were established with anonymized data accessed through the TriNetX platform that included 62,572 patients from the US network and 6377 patients from the EMEA Network. (Additional file 1: Fig. S1). All patients had received treatment for MM. Analyses of the local H12O cohort were performed using IBM SPSS Statistics Version 25. Kaplan‒Meier analysis was used to estimate overall survival, and between-group differences were examined using the log-rank test. All other analyses of the EMEA and US cohorts were performed using the analyses built into the TriNetX platform as previously described [9, 10].

Globally, for patients from H12O, the median OS was 35.61 (28.38–42.84, 95% CI), 55.59 (40.20–70.98, 95%) and 68.67 (54.92–82.42, 95%) months for the 1999–2009, 2010–2014 and 2015–2020 cohorts, respectively (*p* = 0.0001), and the 5-year OS rates were 28%, 43% and 48%, respectively (*p* = 0.0001). Among the group of patients younger than 65 years old, the 5-year OS rates were 42%, 62% and 94%, respectively (*p* = 0.0001). Similarly, for the patients aged between 65 and 75 years old, the OS rates were 27%, 45% and 51%, respectively (*p* = 0.0001). Patients older than 75 years had 5-year OS rates of 9%, 15% and 15%, respectively (*p* = 0.6) (Fig. 1). Among all patients included in the EMEA network, the 5-year OS rates from 1999–2009 versus 2010–2014 were 22.11% versus 35.20% (*p* < 0.0001). The 5-year OS rates from the 2010–2014 versus 2015–2020 time cohorts were 35.20% versus 43.39%, respectively (*p* < 0.0001). Among all patients in the US network, the 5-year OS rates from before 2010 versus 2010–2014 were 56.76% versus 59.24%, respectively (*p* = 0.002). When comparing the 2010–2014 versus 2015–2019 time cohorts, the 5-year OS rates were 59.24% versus 64.83%, respectively (*p* < 0.0001). (Fig. 2). *The results by age group are described in* Additional file 2: Fig. S2 and Additional file 3: Fig. S3*.* In the EMEA cohort, the most commonly used treatment and the most commonly used first-line treatment during all time periods was bortezomib, while lenalidomide is the most commonly used drug in the US cohort, except in the cohort 2010–2014 where bortezomib was the most commonly used first-line treatment. (Additional file 4: Fig. S4).

**Fig. 1:**
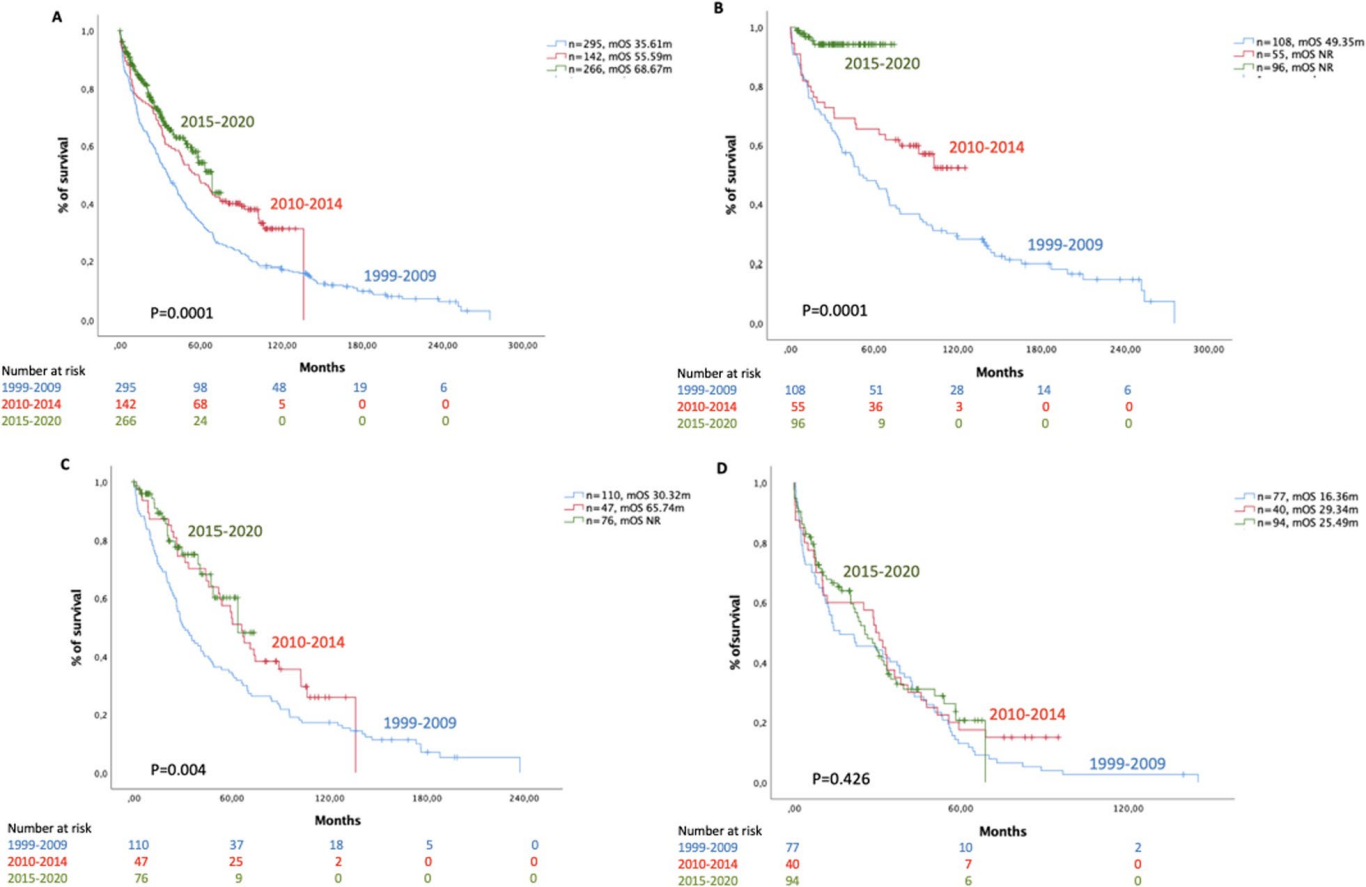
Survival probability of MM patients of H12O cohort. **A** Global cohort. **B** Patients < 65 years. **C** Patients 65–75 years. **D** Patients > 75 years

**Fig. 2:**
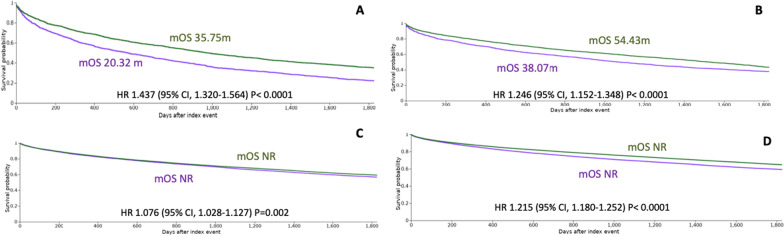
Survival probability of MM patients of EMEA network. **A** Cohort 1999–2010 (purple) versus 2010–2014 (green): all the patients. **B** Cohort 2010–2014 (purple) versus 2015–2020 (green): all the patients. Survival probability of MM patients US network. **C** Cohort 1999–2010 (purple) versus 2010–2014 (green): all the patients. **D** Cohort 2010–2014 (purple) versus 2015–2020 (green): all the patients

In this manuscript, as reported by other studies [11, 12], we identify a relevant increase in the survival of patients with MM over the last 20 years. This could be due to the development in recent decades of new drugs and therapies (mainly proteosome inhibitors, IMIDs and anti-CD38 monoclonal antibodies) for the treatment of patients with MM. However, this increase has only been translated to patients over 75 years; subsequently, MM treatment continues to be a challenge among very elderly patients. This study has some limitations. The data from Trintex platform is not curated, and some bias could be presented. However, the data from our hospital is more refined and with the certainty of proper registration. In conclusion, this large-scale study based on real-world data confirms the previous finding that MM patients have increased their survival in the last two decades in patients younger than 80 years. We have observed heterogeneity in the treatment patterns across different networks and between the USA and Europe. Therefore, these findings reinforce the power of real-world studies based on global federated health research networks to confirm the results of clinical trials.

## Supplementary Information

**Additional file 1: Fig. 1.** Diagram of the different cohorts used in the study.

**Additional file 2: Fig. 2.** Survival probability of MM patients of EMEA network. **A** Cohort 1999–2010 (purple) vs 2010–2014 (green): patients <65 years. **B** Cohort 1999–2010 (purple) vs 2010–2014 (green): patients 65–75 years. **C** Cohort 1999–2010 (purple) vs 2010–2014 (green): patients >75 years. **D** Cohort 2010–2014 (purple) vs 2015–2020 (green): patients <65 years. **E** Cohort 2010–2014 (purple) vs 2015–2020 (green): patients 65–75 years. **F** Cohort 2010–2014 (purple) vs 2015–2020 (green): patients >75 years.

**Additional file 3: Fig. 3.** Survival probability of MM patients of US network. **A** Cohort 1999–2010 (purple) vs 2010–2014 (green): patients <65 years. **B** Cohort 1999–2010 (purple) vs 2010–2014 (green): patients 65–75 years. **C** Cohort 1999–2010 (purple) vs 2010–2014 (green): patients >75 years. **D** Cohort 2010–2014 (purple) vs 2015–2020 (green): patients <65 years. **E** Cohort 2010–2014 (purple) vs 2015–2020 (green): patients 65–75 years. **F** Cohort 2010–2014 (purple) vs 2015–2020 (green): patients >75 years.

**Additional file 4: Fig. 4.** Treatment pathways. **A** EMEA network: 1999–2009. **B** EMEA network: 2010–2014. **C** EMEA network: 2015–2019. **D** US network: 1999–2009. **E** US network: 2010–2014. **F** US network: 2015–2019.

## Data Availability

The data that support the findings of this study is available from the corresponding author upon reasonable request.

## Abbreviations

MM: Multiple myeloma
HCO: Health Care Organizations
