# Correction: Large-scale real-life analysis of survival and usage of therapies in multiple myeloma

**DOI:** 10.1186/s13045-023-01484-8

**Published:** 2023-07-25

**Authors:** N. Lopez-Muñoz, G. Hernández-Ibarburu, R. Alonso, J. M. Sanchez-Pina, R. Ayala, M. Calbacho, C. Cuellar, M. T. Cedena, A. Jiménez-Ubieto, R. Iñiguez, M. Pedrera, J. Cruz, L. Meloni, D. Pérez-Rey, P. Serrano, J. de la Cruz, J. Martinez-Lopez

**Affiliations:** 1 Hematology Department, Hospital 12 de Octubre, CNIO, Complutense University, Madrid, Spain; 2 Biomedical Informatics Group, Universidad Politécnica de Madrid, Madrid, Spain; 3 Data Science Group, Hospital 12 de Octubre, Madrid, Spain; 4 TriNetX Europe NV, Sint-Martens-Latem, Belgium; 5 Research Institute imas12, Hospital 12 de Octubre, Madrid, Spain

**Correction : Journal of Hematology & Oncology (2023) 16:76** **https://doi.org/10.1186/s13045-023-01474-w**

The original article [1] mistakenly truncated co-author, A. Jiménez-Ubieto’s name which has since been amended.
