# Supplementary figures and images for: Large-scale real-life analysis of survival and usage of therapies in multiple myeloma
Source: J Hematol Oncol. 2023 Jul 19;16:76. doi: 10.1186/s13045-023-01474-w (PMC10357768; doi:10.1186/s13045-023-01474-w)

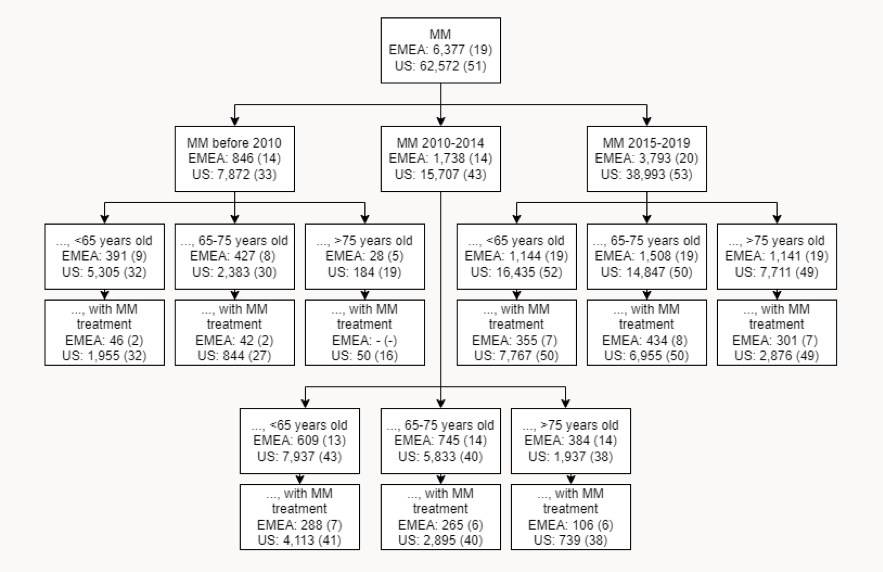

Supplement: Supplementary file 1 — Additional file 1: Fig. 1. Diagram of the different cohorts used in the study. [file 13045_2023_1474_MOESM1_ESM.png]

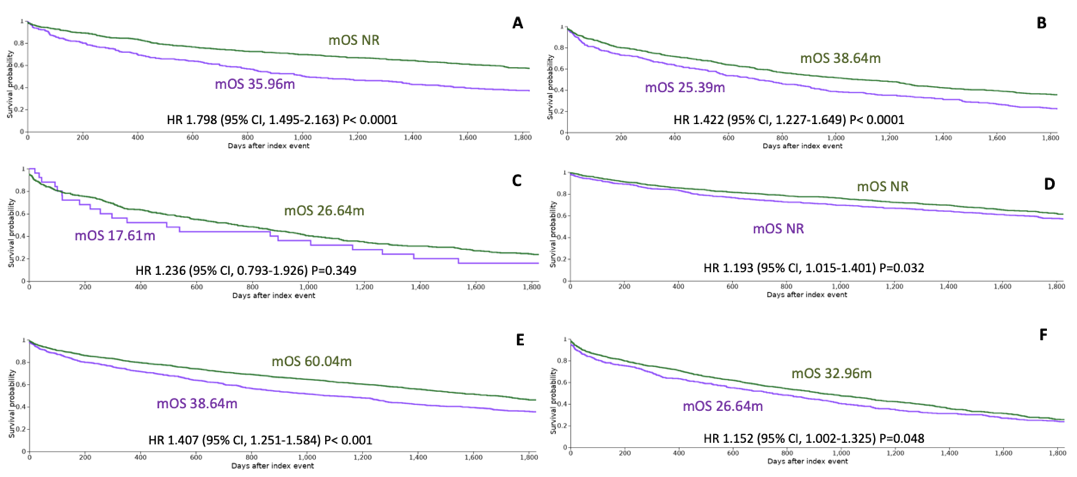

Supplement: Supplementary file 2 — Additional file 2: Fig. 2. Survival probability of MM patients of EMEA network. A Cohort 1999–2010 (purple) vs 2010–2014 (green): patients <65 years. B Cohort 1999–2010 (purple) vs 2010–2014 (green): patients 65–75 years. C Cohort 1999–2010 (purple) vs 2010–2014 (green): patients >75 years. D Cohort 2010–2014 (purple) vs 2015–2020 (green): patients <65 years. E Cohort 2010–2014 (purple) vs 2015–2020 (green): patients 65–75 years. F Cohort 2010–2014 (purple) vs 2015–2020 (green): patients >75 years. [file 13045_2023_1474_MOESM2_ESM.png]

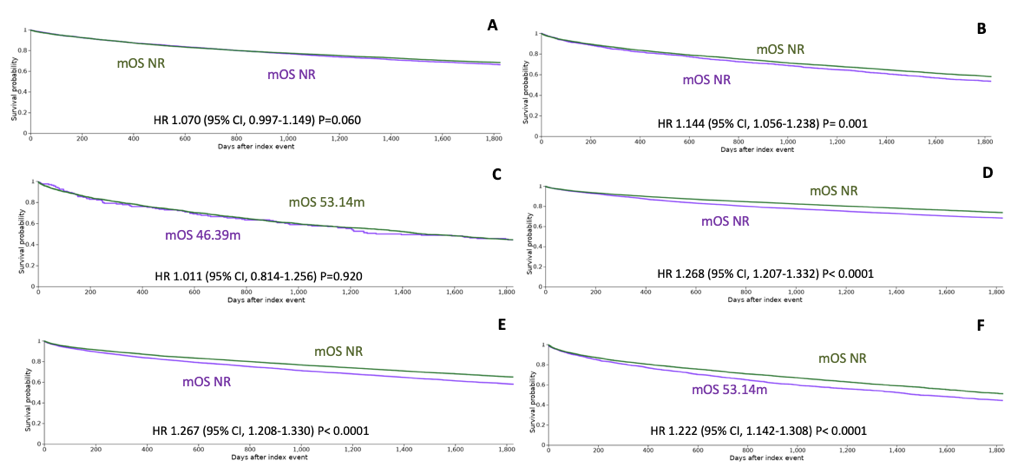

Supplement: Supplementary file 3 — Additional file 3: Fig. 3. Survival probability of MM patients of US network. A Cohort 1999–2010 (purple) vs 2010–2014 (green): patients <65 years. B Cohort 1999–2010 (purple) vs 2010–2014 (green): patients 65–75 years. C Cohort 1999–2010 (purple) vs 2010–2014 (green): patients >75 years. D Cohort 2010–2014 (purple) vs 2015–2020 (green): patients <65 years. E Cohort 2010–2014 (purple) vs 2015–2020 (green): patients 65–75 years. F Cohort 2010–2014 (purple) vs 2015–2020 (green): patients >75 years. [file 13045_2023_1474_MOESM3_ESM.png]

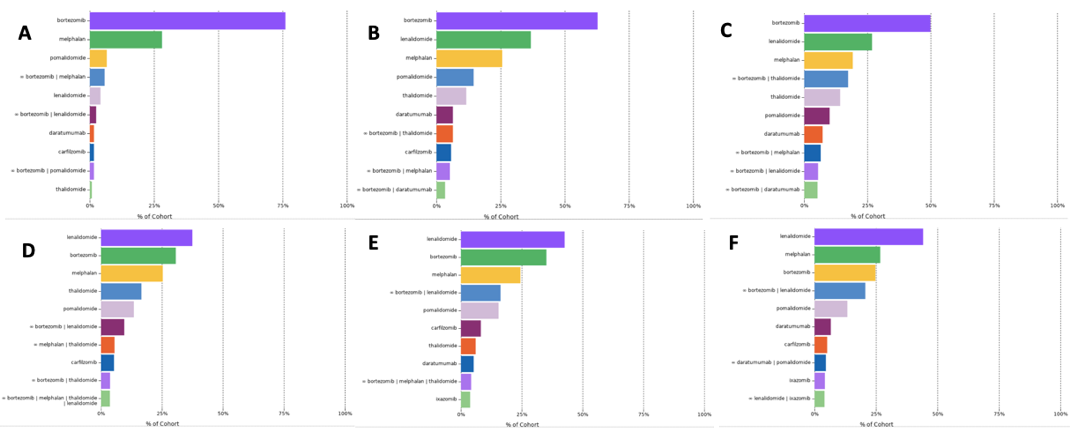

Supplement: Supplementary file 4 — Additional file 4: Fig. 4. Treatment pathways. A EMEA network: 1999–2009. B EMEA network: 2010–2014. C EMEA network: 2015–2019. D US network: 1999–2009. E US network: 2010–2014. F US network: 2015–2019. [file 13045_2023_1474_MOESM4_ESM.png]
